# Supplementary material for: Striatal network modeling in Huntington’s Disease
Source: PLoS Comput Biol. 2020 Apr 17;16(4):e1007648. doi: 10.1371/journal.pcbi.1007648 (PMC7197869; doi:10.1371/journal.pcbi.1007648)
Supplement: Attachment — Submitted filename: resp.pdf [file pcbi.1007648.s001.pdf]

We thank both referees for many useful comments. In general the manuscript has been restructured throughout to take account of these comments. We have therefore not labelled every changed section in colour. However a few sections specifically referred to in our response below are coloured.

Referee 1, response

- My personal opininion is that the ISI statistics is a noisy and very indirect measure to analyze the core phenomenon at work here (the fluctuations of network activity), and it doesn't seem to be an optimal approach to tackle this problem. The logic would have been more convincing if reversed. E.g. constrain the network model on population activity (either a low sampling of their SUA, or the LFP, ...) and then show that they can explain higher order quantities at the single neuron level (e.g. ISI statistics). But this is just a matter of opinion, so if the authors find that tackling this problem through the analysis of the ISI statistics is a good approach, this is fine with me.

  We thank the referee for this suggestion. Unfortunately we do not have simultaneously recorded LFP from most mice in our datasets. However even if we did we believe that to estimate the model on LFP would present many challenges. First LFP recorded in the striatum need not be generated soley by MSN activity, indeed a significant contribution originates from other striatal cells (like FSIs) and it is thought that other strong contributions come from outside the striatum altogether (for example : Carmichael, J. E., Gmaz, J. M., & Van Der Meer, M. A. (2017). Gamma oscillations in the rat ventral striatum originate in the piriform cortex. Journal of Neuroscience, 37(33), 7962-7974.) Second we would need to provide an auxiliary model of how our network model generated membrane potential time series are transformed to LFP, this would introduce further ambiguity and complexity. We also do not have any simulataneously recorded multi-unit activity.

  In fact to some extent we have done the reverse of the referee suggestion. We have estimated the model on properties of the ISI distribution, (its rescaled moments and maximum likelihood parameters for several theoretical distributions) as well as some properties independent or not fully constrained by the ISI distribution (the first two ISI

serial autocorrelations and the local CV distribution (which is different if ISI sequence is shuffled and therefore not equivalent to the ISI distribution)). However we have compared best fit model generated characteristics, in particular the power spectrum, which describes the full temporal structure of the autocorrelation function, with the experimental power spectrum and found remarkable agreement even on timescales much longer than a typical ISI (i.e. the behaviour of the low frequency fluctuations.) Similarly we showed we can also reproduced the slowly decaying character of the ISI serial autocorrelation. In short we demonstrate that tackling the problem using the ISI statistics has turned out to be a highly effective approach.

To explain this a bit better we have completely restructured the first section 'data analysis' of the results.

- How does the observed effect deviates from the experimental variability ? There is no statistical analysis for the observations of line 152-165 and Figure 1 (also Fig. 9). You should plot errorbars on the summary data presented in Figure 1 to show how the observed effect compares to the variability across experiments/mice. The text in the legend: "Bars (exemplar results only for figure clarity) show SEM across the observations in the given datasets." is not clear. What is the "given datasets" here, it should be just the Q175WT dataset, right ? So why "datasets" in plural, is it averaged over datasets ? Just add the variabilty of each datasets in the plot with the N in the legend. Then, you can add panels to emphasize your point if needed.

  We thank the referee for pointing this out. We now have error bars throughout the manuscript in all figures (although for model generated data they are often too small to be seen) on all separate results. This has necessitated breaking some figures up to make more panels for visibility. In particular Fig.2 has now been divided into six panels and we have created a new figure to compare model generated data with experimental data, Fig.9. Since this has also increased the amount of figures we have also removed some results (the combined dataset autocorrelations in Fig.1(b, main panel) of the originally submitted version) since they were less necessary for the main results and to make the presentation a bit shorter. We can put these results back in, or

put them in supplemental material, if necessary according to referee or editorial directions. The results in the original Fig.1(b, inset) are now in Fig.9 of the new version.

We have thoroughly rewritten the description of our experimental datasets in the Methods section, 'Data Analysis', (text coloured red), to explain clearly how many observations are in each dataset and how the error bars are generated. We prefer not to include N in each caption because this information requires some detailed explanation and would be a distraction from the main points. It would also need to be partially repeated in several captions. Instead the explanation for the quantity of observations in each dataset is in the Methods section 'Data Analysis' and the associated figure, Fig.13. Each caption refers to this section.

We have also tried to make our usage of the term 'dataset' clearer by explaining it in the Methods section 'Data Analysis'.

- No "N" in the paper. State the number of animals per genotype in the "Animals" section. I couldn't find the number of animals used, neither in the Methods nor in the Results.

  We have explained this now in the Animals section.

- Is the rather weak age-dependency statistically significant ? From the data of Figure 10, it doesn't seem obvious. I can not see any strong trend in Figure 10F. If this is a weak trend, tone down the importance of this phenomenon (from abstract to discussion).

  We have strongly toned down the age dependency investigation. We have hugely simplified Fig.11 (which was Fig.10 and Fig.11 in the originally submitted manuscript.) The new Fig.11 now only shows results for 3 week interval datasets for YAC and Q175 animals and individual animals for R62 animals. y-axis error bars have been included on all points, which are the fits to the individual datasets. (x-axis error bars in the dataset age are not shown for figure clarity.) The calculation of the error bars is explained in the Methods section 'Data Analysis' (text coloured red). We note the the size of the error bars on the individual datasets is comparable to the size of the fluctuations across the datasets, which suggests they are appropriate. We now only perform

linear regression on these 3 week interval datasets for YAC and Q175 and on individual animals for R62. The slopes obtained from these linear regressions are shown in the Fig.11 insets. Linear regressions were performed using errors in both x and y co-ordinates as explained in the Methods section 'Data Analysis' (text coloured red.) We have removed the Fig.10(E,F) from the originally submitted manuscript altogether.

Although some of the regression slopes appear significant according to our calculations, we have strongly reduced the text in Results related to these age dependent results. We have also removed this from the abstract as the referee suggests.

- The theoretical results should not depend on a specific realisation of the connectivity scheme. A state-of-the-art practice in the analysis of network dynamics in randomly connected assemblies is to run the simulations several times for different seeds generating the random connectivity. Then plot results as mean+/-sd in Figure 4,5,etc... And do the fitting on the mean values not on a single simulation case.

We thank the referee for this point. To do this would be very nice but unfortunately our simulations are very computationally intensive. To generate the 96 simulations used in the model fitting in Fig.6 takes around two months. This is because the simulations are fairly long, 200 seconds, the connectivity is not very sparse (0.2), the network has a quite large number of cells, 2500, and the cell models are detailed including many variables and ion channels and we have integrated them with a small timestep (0.1 ms) to generate precise spike timings. Rather than repeat the same exact same simulation for different seeds, as the referee suggests, we chose to perform simulations more 'densely' at nearby parameter values. Since these simulations were initiated with different random seeds this approach amounts to almost the same thing, since as the simulations approach in parameter space, they would become the same simulation but with different seeds. We believe our choice of parameter increment is reasonable, and the simulations are sufficiently dense in parameter space, because the results shown in Fig.4 vary smoothly with the parameter variations (g_E and g_I). Furthermore these simulations are fairly large, with 2500 cells where each cell is connected to on average 500 others (with an std of 20). Different seeds

would only generate very slightly different networks. Therefore, except possibly very close to the regime transition, different seeds would not result in strong differences in the relevant measured quantities (i.e. the CV, mean ISI etc).

We do note that measurements of the higher order moments of the ISI distribution like the skew, and sometimes the CV, are unstable and can sometimes show quite large fluctuations across simulations when network inhibition, g_I, is close to the network transition regime, and when the driving excitation, g_E, is large. This can be seen in Fig.4(b, blue line) and Fig.4(c) of the originally submitted manuscript, although these results are not included in Fig.4 of the new revised manuscript. These fluctuations cannot be seen in more stable quantities like the firing rate, Fig.4(a), and are much less visible in quantities like the local CV distribution and KS distances, Fig.4(d). These fluctuations in the higher order moments arise because of the presence of very long ISIs which can also occur coherently across cells. These ISIs can be of the order of the simulation time length, 200 secs, and the fluctuations in the higher moments across simulations therefore occur because of the finite observation time of 200 secs. Rather than averaging over different network structures, (much) longer simulations may be more effective in reducing the size of these fluctuations.

The measured quantities shown in Fig.4 and Fig. 8 (i.e. the CV, mean ISI etc) are calculated for each active cell (those which generate more than 10 spikes in a 200 second simulation) and then averaged across each active cell in the simulation. The bars on these figures show the corresponding SEM across active cells. The size of these SEM are also consistent across simulations in general. We do not think that including more simulations with the same parameter settings would change either the mean value shown or the size of the SEM.

We have also clarified in the Methods model description text (coloured red) that the network degree distribution is binomial - i.e the cells are connected at random with probability 0.2. There are no preferential connections in the network structure.

However if the referee insists on this point we would need to request an extension to complete it.

- Figure 1: spreading the labels in all panels make the graphs poorly

readable. Maybe make a legend at the top of the figure so that it's clear that it applies to all panels.

We have fixed legends throughout the manuscript.

- dysregulation, deterministic

  Thank you, we have fixed this.

- We have removed 'dynamically critical' from the abstract

- The text is particularly long ...

  We have removed several results and shortened the paper overall (see below). The paper does remain rather long. However this is because it contains new experimental results, new modeling and a method for comparing model and experimental data, all of which need explaining in sufficient detail.

- Maybe you could use a consistent-color code across figures ...

  We have tried to use a consistent colour code and succeeded to some extent. For example Figs. 2,10,11 describing different phenotype datasets now use a consistent colour scheme. We have only partially used this scheme in Fig.1 and Fig.7 (the WT animals are the same colour as Fig.2,10,11) because these figures turned out to be easier to undestand if different mouse types (YAC, Q175, R62) used the same colour. Fig.8 and Fig.9 also use a consistent scheme for the smaller datasets WT75 and HD12.

Referee 2 response.

- deterministic.

  Here we mean fluctuations are generated by deterministic non-stationary dynamics (like chaos) without stochasticity. We have clarified this in the text (coloured red).

- Fig.4.

  The referee is correct, E is supposed to be g_E. We have fixed this in all figures throughout the manuscript.

- The authors compare the ISI distributions by calculating KS and ISI CV first (Fig. 2 and 4), but this analysis for the data and model are somehow separated. First they examine what distribution fits the experimental data best. Then they do the same for the distribution generated by the model. But in this part there is no direct comparison of the model and data distributions. I dont understand why this is done this way.

  We thank the referee for pointing this issue out.

  In fact we have completely restructured the data analysis section of the results to explain this better, as well as the section comparing best fit model generated and experimentally generated data, 'Comparison of experimental and model generated data'. We use some quantities to fit the data to the model, termed 'features' (for example the maximum likelihood scale parameter of the gamma distribution, the ISI rescaled skew etc) but we also calculate other quantities, like the power spectrum. These other quantities are reported for two reasons - first they are interesting in their own right. For example the consistent way power spectra change (Fig.2) from WT to HD across different types of HD animals has not been noted before. Similarly for the way the KS distance measures change with age consistently across YACWT and Q175Het, Fig.10. Second they provide partially independent means to demonstrate that our model is generating accurate behaviour. We do not fit the whole structure of the power-spectrum, or ISI serial autocorrelation but our model is able to reproduce these characteristics quantitatively, Fig.9.

- Some lengthy explanations are hard to follow. For example, I couldnt follow the long explanations to Fig. 5. There is no conclusion for each of this paragraphs. What are the results following from this data? In general, I would suggest emphasizing a conclusion to each paragraph.

We thank the referee for noting this.

In order to simplify the presentation we have strongly reduced the model description section. From Fig.4 we have removed the description of the skew (panel (d) of original manuscript), we have removed the 'excess KS distance', (panel (c) of the original manuscript.) To make the figures less confusing we have also removed the largest excitation level, g_E=110, results from all four panels, since these results did not add much more explanation than is already shown by the other five levels of g_E. However these simulations at g_E=110 are still used in the model fitting procedure as shown in the heat maps Fig.6.

From Fig.5 we have entirely removed the dependence on g_E. We now only show how the ISI characteristics in Fig.5 depend on g_I. Similarly for the text describing Figs.4,5 we now do not describe in as much detail how ISI characteristics vary with g_E. In fact these descriptions were not necessary for understanding the results in the paper and made it overly complex. We have tried to make the description of network dynamics as brief as possible. However we do require some description of how the network dynamics depends on the parameters in order to demonstrate the presence of the network transition. This is needed to show the most interesting point that the WT animals are close to the transition regime, while HD animals are further from it.

If referee or editor would prefer the previous figures or results to be included we can put these results back the manuscript, or include them as supplemental.

We have also reduced the Discussion section considerably.

- There are no panel labels in fig 6. Probably because of that I couldnt understand what the last two panels of this fig. present.

  We apologize for this. We have fixed panel labels throughout.

- I couldnt understand Fig. 13. What is the horizontal axis? Its also cited right after fig 7, not in the numeric order. I strongly suggesting moving this figure earlier in the paper in the order it was cited.

  We have completely remade this figure so it is clear. It just explains the datasets. Since this is a Methods figure and we do not want to

detract from the main text we have now not referenced it in the main text and left the figure in Methods. The reader is referred to Methods instead of the figure.
