# Supplementary material for: Striatal network modeling in Huntington’s Disease
Source: PLoS Comput Biol. 2020 Apr 17;16(4):e1007648. doi: 10.1371/journal.pcbi.1007648 (PMC7197869; doi:10.1371/journal.pcbi.1007648)
Supplement: Attachment — Submitted filename: resp2.pdf [file pcbi.1007648.s002.pdf]

We thank the referee for many useful comments. It is very important for us to know the interpretations of readers. All changes have been coloured red in the revised version of the manuscript submitted here.

Response to referee

- **The fitting procedure at the core of the study is seemingly still rather unstable and noise-sensitive, so I still struggle to be very confident about its output.**

  We have demonstrated in Fig.14 that, provided the model is correct (i.e. the data is in fact drawn from the model within the explored parameter ranges), the parameters g_I and g_E can be unequivocably determined.

- **Fig.4: provide error bars by bootstrapping either on neurons in the simulated network or time in the simulation.**

  That is subjective. I was asking for the seed variations exactly because I didnt find it smooth enough and because your optimal value for the WT looks slightly like an outlier with respect to the trend of the curve. Also this could help the stability of your inference procedure.

  We have divided 200 second simulations into five 40 second segments. This is described in methods and in the figure 4 caption. For each segment we calculated the mean value of the relevant quantity across cells in the simulation and the error bars show the SEM in this average value across five segments. This does indicate fairly large fluctuations in the mean network CV value across segments, Fig.4(b), in particular in the transition regime g_I $30 - 40$ and for larger g_E. However we do not believe the network simulation used to illustrate the WT at $g_I = 33$, $g_E = 50$ (pink) is an outlier. Indeed while the CV does appear to show a bump at this simulation, many other quantities do not. Several of the KS distances shown in Fig.4(d) do not show a bump. To make this clearer we have included a further quantity which is used as a feature in the model estimation procedure and which is also interesting in its own right. This is the lognormal shape parameter, new Fig.4(c). This quantity does not show any out-of-trend variation at the

simulation used to illustrate the WT, $g_I = 33$, $g_E = 50$, although the simulation at higher $g_E = 75$ (dark green) does in both the CV and the lognormal shape parameter. Indeed we do find quantities utilizing higher moments of the ISI distribution, in particular the skew and to some extent the CV, show larger fluctuations than others. This useful observation is now discussed in the Methods where it clarifies why we have used multiple features in the network estimation procedure. This illustrative WT simulation appears to be an outlier simply because of this fluctuation in the CV trend. We wished to include the ISI CV however because it is an often considered and familiar quantity in neuroscience.

This bump may also reflect the slightly lower than expected (according to the trend) value of the simulation at $g_I = 29$, $g_E = 50$.

We also have to report an error in our previous referee response. When checking these simulations we accidently looked at different code where each simulation is initiated with a different random seed. In fact checking in more detail we now discovered that while simulations at different levels of g_I do indeed have different random seeds, simulations at a given g_I with different g_E are identical. This point is now clearly stated in the Fig.4 caption and in the Methods. This suggests that fluctuations are not solely caused by different seeds since all curves at given g_E in Fig.4 have the same set of seeds. As can been seen the pink and dark green CV curves in Fig.4(b) show quite large fluctuations in trend as g_I is varied, but the cyan curve, for example, does not.

Although many quantities (e.g. the lognormal shape parameter Fig.4(c)) do not show large fluctuations in trend we do agree with the referee that fluctuations in the more unstable CV could be reduced by averaging over more simulations with different seeds. However as explained we would need to request an extension to do this. Furthermore, given the fact that only a small proportion of the quantites used in the model estimation procedure demonstrate large fluctuations, we do not believe our main results would be affected in any way by doing this.

We also point out that the best fit parameters g_E^* and g_I^* for any particular experimental data set are estimated using a weighted average of all network simulations. The single simulation which the referee suggests looks like an outlier is only used as an illustration of how well a partic-

ular single network simulation approximates a particular experimental data set in Fig.8 and Fig.9. The 'optimal' value the referee refers to is not the 'optimal value for the WT', but only the best fit simulation for the particular experimental 3-week dataset labelled 'WT75' with mean age around 75 weeks.

- **There are huge variations in conductance estimate for closely related subjects (of neighboring weeks) in Figure 11, this suggests that you conductance inference is very sensitive to tiny variations in experimental data and therefore not particularly trustworthy. If that's not seed variation, it could be something else, but anything that would stabilize the inference would bring confidence in the method.**

  We have demonstrated in Fig.14 that, provided the model is correct the parameters g_I and g_E can be uniquely and unequivocally determined from the single unit ISI statistics. This is discussed a little more in Discussion now (which is already long). The conductance estimates do show large fluctuations both within age group and between age group in Fig.11. This is because the variations in experimental data are not 'tiny' but can be large. The SEM in the experimental data features used to estimate the model for the two 3-week interval age groups used to illustrate the model fit are shown by the errors on the solid bars in Fig.8. For example, while the WT mean firing rate is around 5 Hz the SEM indicate an error in this mean estimate of over 1 Hz. Fluctuations in firing rate across the experimental spike train observations are correspondingly large. These recordings in any age group are from different animals which are not genetically identical or even littermates or necessarily subject to identical handling or experience from birth, so a range of ISI characteristics is not surprising. Moreover even if the animals were clones with identical experience, our model shows that in any given simulation there are also very large fluctuations in firing rate and all other observables across cells (in the model transition regime). Therefore large fluctuations in the experimental data (given many of the three week experimental datasets include only tens or sometimes hundreds of 200 second spike trains) should be expected from the model. Since our estimation procedure is based on the KL distance between model and experimental distributions of measured feature quantities, there can be several network simulations with sim-

ilar KL distance from the experimental data, resulting in fluctuations in conductance estimates.

- **Fig. 11: indicate which regressions are statistically significant**

  Although some of the regression slopes appear significant according to our calculations And you do not find worth it to share this information ?? As readers, we are looking for quantitative conclusions about the phenomenon studied. Despite my previous comment you still do not provide any answer. If it is significant we want to know it. If it is not (either because there is no trend or because the fitting it too unstable), we also want to know it !

  We have now included p-values calculated using the standard method. This is explained in the Fig.11 caption and in Methods and related the text in Results has been adjusted appropriately. The p-values are included in the Fig.11 caption. We did not indicate the p-values actually in the figure panels because they are already cluttered.

- **clarify in the Discussion the limitation of the ISI approach for inferring network states**

  In short we demonstrate that tackling the problem using the ISI statistics has turned out to be a highly effective approach. I am still not convinced that you can make any claim on network dynamics by only looking at ISI statistics. Network activity fluctuations can be virtually independent from the ISI distributions, because they are crucially constrained by the network synchrony (see e.g. Brunel JCNS 2000 for sets of regimes where either regular or irregular ISI can lead to drastically different network regimes because of different network synchrony). There is nothing in your minimization procedure that relies on an estimate of the synchrony, so I can not see convincing evidence that the network regimes displayed by the model are physiologically relevant. ..... To my opinion this is still a major weakness of the approach, it was rather superficially treated in this revision. The minimum would have been to clearly state and highlight this important limitation in the discussion.

We have added a paragraph in the Discussion addressing this. We are not suggesting that spike times are synchronized across cells, only that slowly varying fluctuations in rates occur coherently across cells. In reality striatal MSNs do show such coherent bursting activity in-vitro and in-vivo (see many references in our paper.) We have simulated a model which also shows coherent activity across cells in physiogically relevant parameter regions. If the model is correct then the network regimes displayed by it are physiologically relevant. We have shown that the single unit data can be used to unambiguously determine the parameters g_I and g_E provided the model is correct and given the other cellular and synaptic parameters are appropriate. Provided the model is correct, this also demonstrates the cells must be firing coherently, since we do not find a regime where cells have the same ISI statistics but do not fire coherently. In the current model it is difficult to imagine how slowly varying fluctuations in input current could be created without coherent fluctuations across cells. In the current model, regimes of synchronous spiking can be found, but these are far outside the physiologically relevant regime and furthermore they do not show ISI statistics similar to the data. That does not mean that there are not models which could show two states : coherent and not-coherent, with indistinguishable single unit ISI characteristics between the two states, but this model does not show such behaviour in the physiological relevant regimes we have investigated.

- **To support this impression, the WT network is said to display Up and Down States (line 574, N.B. no quantification), personally I see the alternation of opposite activations and suppressions in two subpopulations. Why should it be Up and Down states ? (The Vm in *all* neurons should display the same slow oscillation, that wouldnt be the case here).**

  We have moved the mention of up-down states to the Discussion and included the caveats noted by the referee. We feel this is a relevant point which should be made in Discussion.
